# Association between [^68^Ga]NODAGA-RGDyK uptake and dynamics of angiogenesis in a human cell-based 3D model

**DOI:** 10.1007/s11033-021-06513-8

**Published:** 2021-07-02

**Authors:** Maria Grönman, Olli Moisio, Xiang-Guo Li, Tarja Toimela, Outi Huttala, Tuula Heinonen, Juhani Knuuti, Anne Roivainen, Antti Saraste

**Affiliations:** 1 Turku PET Centre, University of Turku, Turku, Finland; 2 FICAM, Tampere University, Tampere, Finland; 3 Turku PET Centre, Turku University Hospital, Turku, Finland; 4 Turku Center for Disease Modeling, University of Turku, Turku, Finland; 5 Heart Center, Turku University Hospital, University of Turku, Hämeentie 11, 20520 Turku, FI Finland

**Keywords:** Angiogenesis, Positron emission tomography

## Abstract

Radiolabeled RGD peptides targeting expression of α_v_β_3_ integrin have been applied to in vivo imaging of angiogenesis. However, there is a need for more information on the quantitative relationships between RGD peptide uptake and the dynamics of angiogenesis. In this study, we sought to measure the binding of [^68^Ga]NODAGA-RGDyK to α_v_β_3_ integrin in a human cell-based three-dimensional (3D) in vitro model of angiogenesis, and to compare the level of binding with the amount of angiogenesis. Experiments were conducted using a human cell-based 3D model of angiogenesis consisting of co-culture of human adipose stem cells (hASCs) and of human umbilical vein endothelial cells (HUVECs). Angiogenesis was induced with four concentrations (25%, 50%, 75%, and 100%) of growth factor cocktail resulting in a gradual increase in the density of the tubule network. Cultures were incubated with [^68^Ga]NODAGA-RGDyK for 90 min at 37 °C, and binding of radioactivity was measured by gamma counting and digital autoradiography. The results revealed that tracer binding increased gradually with neovasculature density. In comparison with vessels induced with a growth factor concentration of 25%, the uptake of [^68^Ga]NODAGA-RGDyK was higher at concentrations of 75% and 100%, and correlated with the amount of neovasculature, as determined by visual evaluation of histological staining. Uptake of [^68^Ga]NODAGA-RGDyK closely reflected the amount of angiogenesis in an in vitro 3D model of angiogenesis. These results support further evaluation of RGD-based approaches for targeted imaging of angiogenesis.

## Introduction

Integrins are transmembrane glycoprotein receptors that mediate interactions between cells and their surroundings during normal development, and in response to physiological and pathophysiological signals. Integrin α_v_β_3_ plays a key role in angiogenesis associated with various disease states, such as ischemic heart disease and during tumor growth [1, 2]. Expression of integrin α_v_β_3_ is generally low in endothelial cells but rises during angiogenesis [1], when the receptor also undergoes a conformational change into the active form [3].

Integrin specific ligands containing the tripeptide sequence arginine–glycine–aspartic acid (RGD) have been radiolabeled for non-invasive imaging of integrin expression using single-photon emission computed tomography (SPECT) or positron emission tomography (PET) [4]. Radiolabeled peptides that selectively bind to α_v_β_3_ integrin have shown promise for non-invasive imaging of pathological conditions associated with angiogenesis, such as peripheral artery disease, ischemic myocardial injury, stroke and cancer [5–8]. In such studies, the uptake of RGD-based tracers has been associated with histological evidence of angiogenesis. However, some studies have suggested that uptake reflects other processes, such as inflammation [9, 10] and fibrosis [11, 12]. Thus, further evaluation of the relationship between the uptake of RGD-based tracer and dynamics of angiogenesis is needed to understand whether the activity of angiogenesis can be accurately quantified by imaging.

[^68^Ga]NODAGA-RGDyK is a novel ^68^Ga-labeled cyclic 1,4,7-triazacyclononane-1-glutaric acid-4,7-diacetic acid-conjugated RGD peptide designed for PET imaging of α_v_β_3_ integrin expression. It has favorable biokinetics, radiation dose, and safety profile [13, 14]. In a previous study using [^68^Ga]NODAGA-RGDyK, we detected an increase in myocardial α_v_β_3_ integrin expression in ischemic myocardium, localized within irreversibly injured myocardium, in the absence of an increase in angiogenesis [12]. To further evaluate the ability of [^68^Ga]NODAGA-RGDyK to measure angiogenic activity, we investigated whether binding of this agent reflects the amount of angiogenesis in a human cell-based 3D in vitro model of angiogenesis [15, 16]. This model consisting of a co-culture of human umbilical cord vein endothelial cells (HUVECs) and human adipose stromal cells (hASCs) mimics dynamics of the development of normal human vasculature. After stimulation with growth factors, the cells produce a vascular-like tubule network with morphology of mature vessels within few days. The density of the vascular network can be reproducibly controlled by adjusting concentrations of growth factors.

## Materials and methods

### Angiogenesis tissue model

We used a human cell-based 3D model of angiogenesis developed at the Finnish Centre for Alternative Methods (FICAM) [15, 16]. When establishing the cell model, hASC cells were thawed, propagated for 9 days, and then seeded onto a plastic coverslip in 24-well plates (for receptor binding studies) or in 2-well chamber microscope slides (for autoradiography studies). On the same day, HUVECs that had been thawed and propagated for 3 days were plated on top of the hASC cell layer to form a co-culture. The next day, stimulation of the vascular-like tubule network formation was initiated by increasing the concentration of vascular endothelial growth factor (VEGF) (R&D Systems, Inc, Minneapolis, US) and fibroblast growth factor β (FGF-β) (R&D Systems, Inc, Minneapolis, US); induction medium was replaced with fresh medium after 3 days. To obtain a gradient in tubule network density, four different growth factor concentrations were used: 10 ng/ml VEGF and 1 ng/ml FGF-β (defined as 100%), and 25%, 50%, and 75% of that maximum concentration. Co-culture of hASCs and HUVECs without induction of a tubule network (because inductive growth factors were not added) served as a control. Cell culture was terminated 6 days after the start of the tubule network stimulation.

### Radiochemistry

The tracer precursor, 1,4,7-triazacyclononane-1-glutaric acid-4,7-diacetic acid-conjugated RGD peptide (cyclo[L-arginylglycyl-L-α-aspartyl-D-tyrosyl-N6-([[4,7-bis(carboxymethyl)-1,4,7-triazonan-1-yl]acetyl])-L-lysyl]; NODAGA-RGDyK), was purchased from ABX Advanced Biochemical Compounds GmbH (product number 9805; Radeberg, Germany). ^68^Ga was obtained from a ^68^Ge/^68^Ga generator (Eckert & Ziegler, Valencia, CA, USA) by elution with 0.1 M aqueous hydrochloric acid. ^68^Ga-eluate (500 μl) was mixed with sodium acetate (18 mg) to a pH of approximately 5. NODAGA-RGDyK (10 nmol, dissolved in deionized water to generate a stock solution of 1 mM) was added, and the reaction mixture was heated at 100 °C for 15 min. No further purification was performed. The radiochemical purity of [^68^Ga]NODAGA-RGDyK was determined by reversed-phase high-performance liquid chromatography (HPLC) on a Jupiter C18 column (4.6 × 150 mm, 300 Å, 5 μm; Phenomenex, Torrance, CA, USA). HPLC conditions were as follow: flow rate = 1 ml/min, λ = 220 nm. Gradient system: A = 0.1% trifluoroacetic acid (TFA) in water; B = 0.1% TFA in acetonitrile. A/B gradient: 0–5 min 97/3, and 5–15 min from 97/3 to 0/100. The HPLC system consisted of a LaChrom instrument (Hitachi; Merck, Darmstadt, Germany) coupled with a flow-through radiomatic 150TR radioisotope detector (Packard, Meriden, CT, USA). At the end of the syntheses, radiochemical purity and molar activity of [^68^Ga]NODAGA-RGDyK were ≥ 95% and 11.9 ± 5.9 GBq/µmol, respectively.

### In vitro binding of [^68^Ga]NODAGA-RGDyK

Transport medium was removed from the wells, and tissue cultures were incubated in medium for 1 h at 37 °C. The medium was Dulbecco’s modified Eagle’s medium/Nutrient Mixture F-12 (DMEM/F12) supplemented with 2.56 mM L-glutamine, 0.1 nM 3,3’,5-triiodo-L-thyronine sodium salt, 6.65 µg/ml insulin, 6.65 µg/ml transferrin, 6.65 ng/ml selenious acid, 1% bovine serum albumin (BSA), 2.8 mM sodium puryvate, 200 µg/ml ascorbic acid, 0.5 µg/ml heparin, 2 µg/ml hydrocortisone, 10 ng/ml VEGF, and 1 ng/ml FGF-β. Then, the medium was removed, and [^68^Ga]NODAGA-RGD in phosphate-buffered saline (PBS, pH 7.4) was added and incubated for 90 min in 37 °C. Incubation time and temperature were chosen according to a previous study [17]. After the tracer was removed, the cells were washed once with PBS. The cover slips that contained the cells were measured for radioactivity using a gamma counter (Wizard 3″; PerkinElmer/Wallac, Turku, Finland).

### In vitro autoradiography

Transport medium was removed from the chamber slides, and warm medium was added. Tissue cultures were incubated for 1 h at 37 °C; medium was removed; and [^68^Ga]NODAGA-RGDyK was added. After incubation for 90 min at 37 °C, the tracer solution was removed, the chamber walls were removed, and the cells were washed twice with PBS. Slides were air dried for 10 min and exposed on a phosphorimaging plate (BAS-TR2025; Fuji Photo Film, Tokyo, Japan) for 1 h. The distribution of radioactivity on the plate was visualized and quantified using a Fluorescent Image Analyzer (Fujifilm FLA-5100, Fuji Photo Film) and the AIDA Image analyzer software v.4.55 (Raytest Isotopenmessgeräte GmbH, Straubenhardt, Germany). Autoradiography uptake was expressed as photostimulated luminescence per square millimeter (PSL/mm^2^), and the vessel-to-base cell layer ratio was calculated by dividing the uptake of the vessels by the uptake of the base cell layer consisting of hASCs and HUVECs without growth factors.

### Immunohistochemistry

After measurements with gamma counter or autoradiography, the tissues were fixed in 70% ethanol for 15 min and stained with antibodies against von Willebrand factor (vWF) and collagen IV. Cells were permeabilized with Triton X-100 for 15 min, and nonspecific binding sites were blocked with 10% BSA for 30 min. Primary antibodies (anti-von Willebrand factor, dilution 1:100; anti-collagen IV, dilution 1:500; Sigma-Aldrich, Saint Louis, Missouri, US) were applied overnight. After washing steps, secondary antibodies (tetramethylrhodamine [TRITC]-conjugated anti-rabbit for von Willebrand factor and fluorescein isothiocyanate [FITC]-conjugated anti-mouse for collagen IV; Sigma-Aldrich, Saint Louis, Missouri, US) were applied for 45 min. Secondary antibodies were washed away, and PBS was left in the wells. Fluorescence imaging of stained tubular structures was performed using a Cell-IQ automated image analysis system (CM-Technologies, Tampere, Finland). The degree of angiogenesis in each image was graded manually; the scoring criterion was the intensity of the vascular-like tubular network (tubule length and branching). The highest score was 5 (dense, highly branched tubular network), and the lowest score was 0 (no or minor tubules). In order to detect α_v_β_3_ integrin, tissues were stained with anti-integrin α_v_β_3_ antibodies (dilution 1:100, Sigma-Aldrich, Saint Louis, Missouri, US) and Alexa Fluor 488 conjugated anti-mouse secondary antibody.

### Statistical analyses

SPSS Statistics software v.25 (IBM, Armonk, NY, USA) was used for statistical analyses. Unpaired Student’s t-test was used for comparisons between two groups, and comparisons between multiple groups were performed using ANOVA with Tukey’s post hoc test. Spearman’s rank test (r_s_) was used to analyze correlation. p < 0.05 was considered statistically significant.

## Results

### In vitro tracer binding

**Fig. 1:**
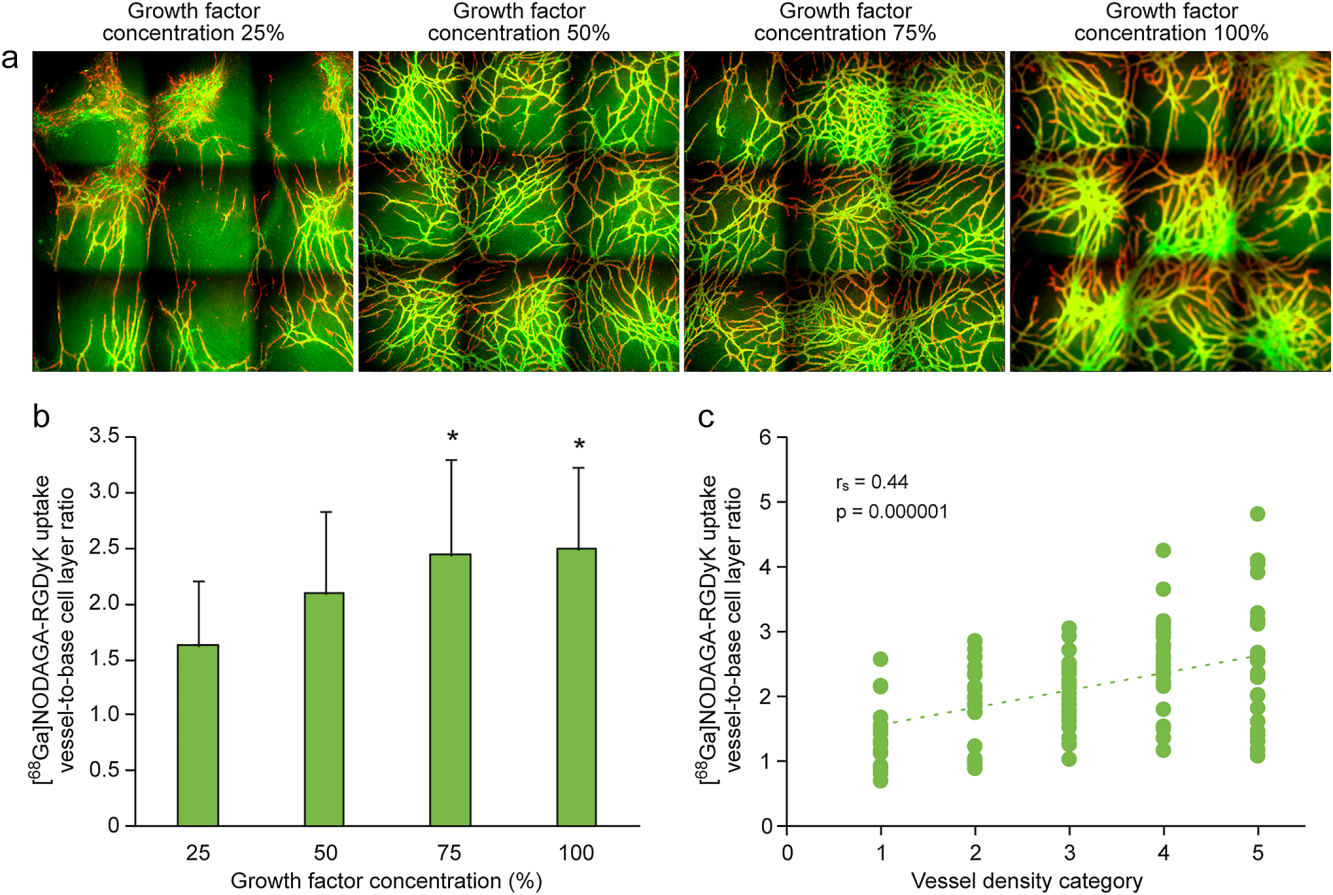
Representative image of vascular-like tubule networks with different growth factor concentrations of 25%, 50%, 75%, and 100% stained for von Willebrand factor (red) and collagen IV (green) (**a**). The graph in **b** shows the vessel-to-base cell layer ratio of [^68^Ga]NODAGA-RGDyK uptake at the indicated growth factor concentrations, and the graph in **c** shows the correlation between vessel density, and the ratio of [^68^Ga]NODAGA-RGDyK uptake between vessel and base cell layer. Asterisks denote a statistically significant difference relative to a growth factor concentration of 25%. (Color figure online)

We performed a tracer binding study in a human cell-derived angiogenesis tissue model using four different concentrations of angiogenesis-promoting growth factors, which yielded vascular-like tubule networks of four different densities. The density of tubules gradually increased with growth factor concentration (Fig. 1a). Relative to control cells that were not treated with growth factors, uptake of [^68^Ga]NODAGA-RGDyK was 1.6-fold higher at 25% growth factor concentration, 2.1-fold higher at 50%, 2.4-fold higher at 75%, and 2.5-fold higher at 100%. Relative to the 25% concentration, uptake of [^68^Ga]NODAGA-RGDyK was significantly higher at 75% (p = 0.0003) and 100% (p = 0.0002) (Fig. 1b). [^68^Ga]NODAGA-RGDyK uptake also correlated with the density of vascular-like tubule network, as determined by visual examination of histological staining (Fig. 1c).

### Autoradiography

**Fig. 2:**
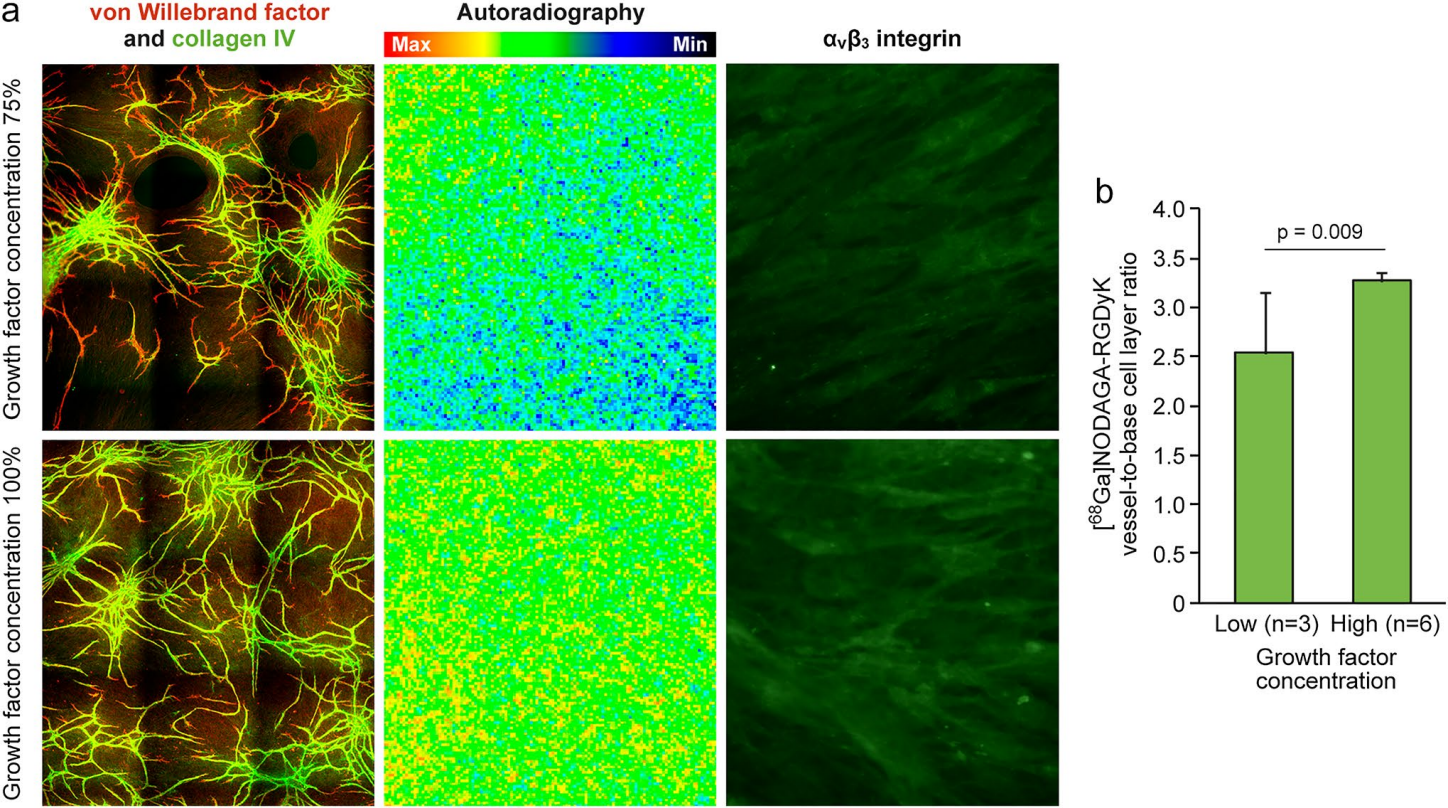
Examples of immunohistochemistry and autoradiography at growth factor concentrations of 75% and 100% (**a**). Tubules were stained for von Willebrand factor (red) and collagen IV (green) in the double-stained images, and for α_v_β_3_ integrin (green). Panel **b** shows [^68^Ga]NODAGA-RGDyK uptake in tissue at growth factor concentrations of 75% and 100%. (Color figure online)

Representative autoradiographs of [^68^Ga]NODAGA-RGDyK uptake in vessels with growth factor concentrations of 75% and 100% are shown in Fig. 2. Uptake was localized in the regions of angiogenesis, indicated by tubule structures positive for vWF and collagen IV, and was significantly higher at 100% vs. 75% growth factor concentration (vessel-to-base cell layer ratio: 3.3 ± 0.09 vs. 2.5 ± 0.63; p = 0.009).

## Discussion

In this study, using an in vitro 3D model of angiogenesis, we found that [^68^Ga]NODAGA-RGDyK binding was proportional to the amount of angiogenesis. Our experiments were performed in a human cell-based angiogenesis tissue model consisting of a co-culture of HUVECs and human adipose stromal cells (hASCs), which generate a reproducible, extensive, mature vascular-like tubule network after stimulation with a growth factor cocktail [15, 16]. This is the first study of an RGD targeting tracer in a tissue model that mimics post-infarct angiogenesis. The density of the tubule network increased with the concentration of growth factors. Vessels in this model exhibit mature properties and become surrounded by pericytes [16, 18]. Pericyte coverage is a characteristic of maturation of newly formed vessels in the myocardium after infarction [2], indicating that the model we used accurately represents in vivo angiogenesis. α_v_β_3_ integrin also seems to be involved in the maturation process, as evidenced by the fact that anti-α_v_β_3_ integrin therapy decreases pericyte coverage in tumor vessels [19]. Our results lend support to the concept that [^68^Ga]NODAGA-RGDyK can be used to quantitatively assess activity of angiogenesis, which is of particular importance for predicting and monitoring therapy responses.

Our results are in line with previous studies showing that the uptake of integrin-targeting RGD peptides is associated with histological evidence of newly formed blood vessels [6], coincides with hypoxia [20], and can be modified by therapy inducing [21, 22] or inhibiting angiogenesis [23]. However, the limited number of time points over prolonged period (for example from 3 days to 3 months after an ischemic insult) does not permit detailed evaluation of association between tracer uptake and ongoing angiogenesis. Furthermore, in addition to mediating angiogenesis, α_v_β_3_ integrin also regulates macrophage inflammatory responses and myofibroblast differentiation [24, 25]. Therefore, the uptake of tracers targeting α_v_β_3_ integrin may not merely represent angiogenesis, but also other events during the repair process after ischemic myocardial injury. Because multiple cell types express α_v_β_3_ integrin, in vivo studies measuring RGD uptake in the context of angiogenesis have yielded conflicting results, as angiogenesis does not always co-localize with integrin expression [9–12]. Studied RGD-based peptides also have different selectivity for different types of integrins [26]. Our in vitro 3D model of angiogenesis provides a more controlled and isolated environment for newly formed vessels that lacks most of the other α_v_β_3_ integrin binding cell types, such as myofibroblasts and macrophages, which are present in in vivo models of myocardial ischemia and infarction. This enables specific evaluation of angiogenesis-associated α_v_β_3_ integrin expression using α_v_β_3_ integrin selective tracer [^68^Ga]NODAGA-RGDyK.

Uptake of [^68^Ga]NODAGA-RGDyK in vessels was elevated in the presence of higher growth factor concentrations and correlated with the abundance of the vessels. Due to problems with tissue attachment on slides at lower concentrations of growth factors, we performed autoradiography analysis only on the slides treated with concentrations of 75% and 100%; this represents a limitation of the study. However, we were able to detect a difference in [^68^Ga]NODAGA-RGDyK binding between the two concentrations measured. Resolution of autoradiography did not allow detailed localization of the tracer signal with individual vessel structures. For this purpose, a different probe including an optical label could be used in experimental setting instead of a [^68^Ga]NODAGA-RGDyK available for clinical use. Finally, detection of angiogenesis with RGD based tracers is dependent on integrin expression. Although α_v_β_3_ integrin is typically expressed during angiogenesis, it may not always be present [27] and thus, there may not be tracer uptake under such circumstances.

## Conclusions

[^68^Ga]NODAGA-RGDyK correlated with the amount of angiogenesis in a human cell-based in vitro model of angiogenesis indicating that the tracer uptake reflects ongoing angiogenic activity. These results support further evaluation of RGD-based approaches for targeted imaging of angiogenesis in various disease states, such as ischemic heart disease and cancer. However, the possibility of nonspecific binding should be taken into account when performing in vivo imaging studies of angiogenesis.

## References

[1] Brooks PC, Clark RA, Cheresh DA (1994). Requirement of vascular integrin alpha v beta 3 for angiogenesis. Science.

[2] Simons M, Alitalo K, Annex BH (2015). State-of-the-art methods for evaluation of angiogenesis and tissuevascularization: a scientific statement from the American heart association. Circ Res.

[3] Mahabeleshwar GH, Chen J, Feng W (2009). Integrin affinity modulation in angiogenesis. Cell Cycle.

[4] Haubner R, Maschauer S, Prante O (2014). PET radiopharmaceuticals for imaging integrin expression: tracers in clinical studies and recent developments. Biomed Res Int.

[5] Sun ZY, Zhu Y (2014). Application of (68)Ga-PRGD2 PET/CT for αvβ3-integrin imaging of myocardial infarction and stroke. Theranostics.

[6] Dobrucki LW, Tsutsumi Y, Kalinowski L (2010). Analysis of angiogenesis induced by local IGF-1 expression after myocardial infarction using microSPECT-CT imaging. J Mol Cell Cardiol.

[7] Jenkins WSA, Vesey AT, Stirrat C (2016). Cardiac α _V_ β _3_ integrin expression following acute myocardial infarction in humans. Heart.

[8] Beer AJ, Schwaiger M (2008). Imaging of integrin αvβ3 expression. Cancer Metastasis Rev.

[9] Laitinen I, Saraste A, Weidl E (2009). Evaluation of αvβ3 integrin-targeted positron emission tomography tracer 18F-galacto-RGD for imaging of vascular inflammation in atherosclerotic mice. Circ Cardiovasc Imaging.

[10] Eo JS, Paeng JC, Lee S (2013). Angiogenesis imaging in myocardial infarction using 68Ga-NOTA- RGD PET: characterization and application to therapeutic efficacy monitoring in rats. Coron Artery Dis.

[11] van den Borne SWM, Isobe S, Verjans JW (2008). Molecular imaging of interstitial alterations in remodeling myocardium after myocardial infarction. J Am Coll Cardiol.

[12] Grönman M, Tarkia M, Kiviniemi T (2017). Imaging of αvβ3 integrin expression in experimental myocardial ischemia with [68Ga]NODAGA-RGD positron emission tomography. J Transl Med.

[13] Buchegger F, Viertl D, Baechler S (2011). 68Ga-NODAGA-RGDyK for αvβ3 integrin PET imaging. Nuklearmedizin.

[14] Gnesin S, Mitsakis P, Cicone F (2017). First in-human radiation dosimetry of 68Ga-NODAGA-RGDyK. EJNMMI Res.

[15] Toimela T, Huttala O, Sabell E (2017). Intra-laboratory validated human cell-based in vitro vasculogenesis/angiogenesis test with serum-free medium. Reprod Toxicol.

[16] Huttala O, Vuorenpää H, Toimela T (2015). Human vascular model with defined stimulation medium—a characterization study. ALTEX.

[17] Knetsch PA, Petrik M,  Griessinger CM (2011). [68Ga]NODAGA-RGD for imaging αvβ3 integrin expression. Eur J Nucl Med Mol Imaging.

[18] Sarkanen JR, Mannerström M, Vuorenpää H (2011). Intra-laboratory pre-validation of a human cell based in vitro angiogenesis assay for testing angiogenesis modulators. Front Pharmacol.

[19] Reinmuth N, Liu W, Ahmad SA (2003). αvβ3 integrin antagonist S247 decreases colon cancer metastasis and angiogenesis and improves survival in mice. Cancer Res.

[20] Kalinowski L, Dobrucki LW, Meoli DF (2008). Targeted imaging of hypoxia-induced integrin activation in myocardium early after infarction. J Appl Physiol.

[21] Cai M, Ren L, Yin X (2016). PET monitoring angiogenesis of infarcted myocardium after treatment with vascular endothelial growth factor and bone marrow mesenchymal stem cells. Amino Acids.

[22] Johnson LL, Schofield L, Donahay T (2008). Radiolabeled arginine-glycine-aspartic acid peptides to image angiogenesis in swine model of hibernating myocardium. JACC Cardiovasc Imaging.

[23] Kazmierczak PM, Todica A, Gildehaus FJ (2016). 68Ga-TRAP-(RGD)3 hybrid imaging for the in vivo monitoring of αvβ3-integrin expression as biomarker of anti-angiogenic therapy effects in experimental breast cancer. PLoS ONE.

[24] Antonov AS, Antonova GN, Munn DH (2011). αVβ3 integrin regulates macrophage inflammatory responses via PI3 kinase/Akt-dependent NF-κB activation. J Cell Physiol.

[25] Sarrazy V, Koehler A, Chow ML (2014). Integrins αvβ5 and αvβ3 promote latent TGF-β1 activation by human cardiac fibroblast contraction. Cardiovasc Res.

[26] Kapp TG, Rechenmacher F, Neubauer S (2017). A comprehensive evaluation of the activity and selectivity profile of ligands for RGD-binding integrins. Sci Rep.

[27] Hynes RO (2002). A reevaluation of integrins as regulators of angiogenesis. Nat Med.

